# Individual Differences in Interoceptive Accuracy Are Correlated With Salience Network Connectivity in Older Adults

**DOI:** 10.3389/fnagi.2020.592002

**Published:** 2020-12-01

**Authors:** Daisuke Ueno, Teruyuki Matsuoka, Yuka Kato, Nobutaka Ayani, Saaya Maeda, Minato Takeda, Jin Narumoto

**Affiliations:** Department of Psychiatry, Graduate School of Medical Science, Kyoto Prefectural University of Medicine, Kyoto, Japan

**Keywords:** interoception, insular cortex, salience network, heartbeat counting task, posterior–anterior shift in aging

## Abstract

Interoceptive accuracy refers to the ability to consciously perceive the physical condition of the inner body, including one’s heartbeat. In younger adults, interoceptive accuracy is correlated with insular and orbitofrontal cortical connectivity within the salience network (SN). As interoceptive accuracy and insular cortex volume are known to decrease with aging, we aimed to evaluate the correlation between SN connectivity and interoceptive accuracy in older adults. 27 older adults (mean age, 77.29 years, SD = 6.24; 19 female) underwent resting-state functional magnetic resonance imaging, followed by a heartbeat counting task and neuropsychological test. We evaluated the correlation between interoceptive accuracy and SN connectivity with age, sex, cognitive function, and total gray matter volume as covariates. Region of interest-to-region of interest analyses showed that interoceptive accuracy was positively correlated with the functional connectivity (FC) of the left rostral prefrontal cortex with the right insular, right orbitofrontal, and anterior cingulate cortices [*F*(6,16) = 4.52, false discovery rate (FDR)-corrected *p* < 0.05]. Moreover, interoceptive accuracy was negatively correlated to the FC of the left anterior insular cortex with right intra-calcarine and visual medial cortices (*F*(6,16) = 2.04, FDR-corrected *p* < 0.10). These findings suggest that coordination between systems, with a positive correlation between left rostral prefrontal cortex and the SN and a negative correlation between left insular cortex and vision-related exteroceptive brain regions, is important for maintaining interoceptive accuracy in older adults.

## Introduction

Interoception collectively refers to afferent sensory information arising from the sensation, perception, and awareness of afferent feedback from the viscera, which maintains homeostatic function (Craig, 2002). Interoception is distinguishable from exteroception and proprioception (Sherrington, 1948). Renewed interest in interoception parallels a growing appreciation that cognition is embodied, and that cognitive and emotional processes are biased by extracerebral changes, as captured; for example, in the somatic marker hypothesis (Damasio et al., 1991). Furthermore, interoceptive ability is relevant to psychological construction models of emotion that propose a basis for emotions as the emergent products of psychological ingredients, which are some form of information from the body (primary) and a process by which internal sensory or affective states (secondary) are made meaningful as related to or caused by the external surroundings (Gross and Barrett, 2011).

Heartbeat perception is frequently used in the measurement of interoceptive awareness, as it is simple, practical, and less invasive than other measures. Previous task-related functional magnetic resonance imaging (fMRI) studies have revealed that interoceptive accuracy is correlated with activity in the insular cortex and anterior cingulate cortex (ACC), regardless of the interoceptive task used (Critchley et al., 2004; Schulz, 2016; Stern et al., 2017). Furthermore, resting state fMRI studies, which use the temporal correlation between fluctuations in different areas as a measure of intrinsic functional connectivity (FC), have identified the salience network (SN), involving the bilateral anterior insular cortices and dorsal ACC, as well as the anterior prefrontal cortex (aPFC), supramarginal gyrus (SMG), striatum/basal ganglion, thalamus, and cerebellum, as important for integrating highly processed sensory data with visceral, autonomic, and hedonic markers, to guide the appropriate behavior (Seeley et al., 2007; Guo et al., 2012). Chong et al. (2017) examined the relationship between SN connectivity and interoceptive accuracy in 26 healthy young adults and found a positive correlation between heartbeat counting accuracy and salience network functional connectivity (SN FC) of the right and left posterior insular cortices, but no correlation was found for the FC of the ACC.

Basic processes in numerous sensory modalities, such as exteroception and proprioception, decrease with aging. Numerous studies have shown age-related decreases in primary somatosensory cortex (Good et al., 2001; Sowell et al., 2003; Salat et al., 2004), insular cortex (Good et al., 2001; Resnick et al., 2003), and SN connectivity (Onoda et al., 2012). However, only a few studies have examined the relationship between age and interoception. Although several studies (Khalsa et al., 2009b; Murphy et al., 2017) have reported that age is negatively correlated with interoceptive accuracy, to our knowledge, no study has examined the relationship between SN connectivity and interoceptive accuracy in older adults. Therefore, the current study sought to fill this knowledge gap by examining the correlations between SN connectivity and individual differences in interoceptive accuracy, as assessed by the heartbeat counting task, in a group of older adults. We used the heartbeat counting task, rather than the heartbeat discrimination task, since it is more reflective of the internal monitoring processes (Critchley et al., 2004; Pollatos et al., 2005; Chong et al., 2017). We hypothesized that better performance on the heartbeat counting task would be correlated with greater FC of the SN.

## Materials and Methods

### Participants

Thirty-one older adults (mean age = 77.08, standard deviation SD = 1.19, range = 61.2–89.0; 22 female) were enrolled in the study. However, we excluded the data of four older adults because of a failure to follow the instructions or missing values. Thus, 27 older adults (mean age = 77.30 years, SD = 6.20, range = 61.20–86.90; 19 female) were finally included. 26 participants were right-handed and 1 participant was left-handed. Classification of handedness was based on the modified 25-item version of the Edinburgh Inventory (Oldfield, 1971). In this study, the sample size could not be calculated from the effect size, power, and significance level because of the nature of the MRI imaging analysis (the statistical analysis would be performed on numerous voxels with multiple comparison correction). Therefore, the sample size was determined with reference to Chong et al. (2017).

Participants comprised outpatients of the Center for the Diagnosis of Dementia, Kyoto Prefectural University of Medicine, as well as volunteers who lived locally or were a member of an employment service center for the elderly, who were aged 60 years or more and had maintained activities of daily living. Participants recruited from the Center for the Diagnosis of Dementia included both healthy adults and those with mild cognitive impairment, based on the results of various neuropsychological tests. The exclusion criteria were as follows: dementia or intellectual disability; a history of mental illness; brain injury; drug or alcohol abuse; serious impairment in vision, hearing, or the function of both hands; and unable to undergo MRI scanning. This study was conducted from October 28, 2017 to September 28, 2019, and was approved by the ethics committees of Kyoto Prefectural University of Medicine (ERB-C-853-3). Informed consent for participation was obtained from all participants.

### Procedures

After completing the informed consent process, participants were taken to the MRI scanner at the Kajiicho Medical Imaging Center, where they completed a neuroimaging session (see section “Imaging Acquisition Protocol”). Participants were then escorted back to the university hospital to complete the heartbeat counting task and the Mini-Mental State Examination (MMSE).

### Imaging Acquisition Protocol

The participants underwent one neuroimaging session on a 3T Philips Achieva 3.0 Quasar Dual (Royal Philips, Japan) with a 32-channel head coil. The session comprised a high-resolution T1-weighted three-dimensional magnetization-prepared rapid gradient-echo (3D MPRAGE) at 3.0 T scan of the entire brain in 170 sagittal slices (magnetization prepared rapid gradient echo sequence, with repetition time and echo time = shortest, flip angle = 9°, field of view = 256 × 256 mm^2^, and voxel size = 1.0 × 1.0 × 1.2 mm, slice thickness = 1.2 mm) and a 10-min resting-state fMRI scan (T2×-weighted echo planar sequence, with repetition time = 2500 ms, echo time = 30 ms, flip angle = 80°, 40 axial slices, field of view = 212 × 212 mm^2^, voxel size = 3.31 × 3.31 × 3.20 mm, matrix size = 64, no interslice skip, slice thickness = 3.2 mm, 0.8-mm gap, and interleaved collection), during which participants were instructed to remain awake and fixate on a black cross in the center of a white screen.

### Heartbeat Counting Task

All participants completed a heartbeat counting task immediately after scanning (i.e., on the same day). In accordance with previous work (Schandry, 1981; Pollatos et al., 2005; Meissner and Wittmann, 2011; Kuehn et al., 2016), participants were instructed to attend to their own heartbeat and silently count the number of beats within five intervals (15, 25, 35, 45, and 100 s; one trial each). Since older adults generally have decreased interoceptive accuracy, we included a 15-s interval, allowing us to measure interoceptive accuracy in short to long intervals. The start and end of each counting interval were indicated by the experimenter. At the end of each interval, participants were prompted to indicate the number of heartbeats counted, followed by a 10-s rest period before the start of the next counting interval. Additionally, participants were instructed to refrain from opening their eyes or using any other physical strategies to aid in the counting of heartbeats. Moreover, to confirm a distinction between heartbeat and time counting, participants were instructed to count time silently over the same five intervals (15, 25, 35, 45, and 100 s), immediately after completing all heartbeat counting intervals.

The heartbeats of the participants were continuously monitored throughout the heartbeat counting task using a pulse oximeter (model 9560 Onyx 2, Star Product Ltd., Tokyo, Japan). While it is possible that different results would be observed with a different measure of interoception allowing easier detection of heartbeats, almost all existing studies of the relationship between interoception and aging have employed this task as an objective index of interoceptive accuracy. Using a finger clip pulse oximeter to measure their own pulse is easier than using an electrocardiogram (ECG) pulse oximeter and a hard-clip (but not soft-clip) oximeter, and the ECG pulse oximeter is correlated with increased perception of heartbeat at the finger (Murphy et al., 2019). The average pulse rate was sampled, and participants were given a 5-min rest to stabilize their pulse rate before the task was started.

Interoceptive accuracy scores were calculated using the formula in Pollatos et al. (2005) and Chong et al. (2017); however, we calculated relative differences, rather than absolute differences, due to remaining individual differences in the number of counted heartbeats compared to the recorded number of heartbeats in older adults who overestimated or underestimated the number of heartbeats in a given time interval. For each participant, accuracy in the perception of the heartbeat was quantified by first taking the relative difference between the actual number of heartbeats recorded by the pulse oximeter and the number of heartbeats counted by the participant, divided by the actual number of heartbeats. This value was then subtracted from 1 and averaged across all trials to yield a heartbeat counting score, such that scores near 100 indicate higher accuracy. Moreover, scores less than 100 indicate that the number of counted heartbeats was underestimated (relative to the number of recorded heartbeats). The mathematical formula was, thus, as follows:

$$\frac{1}{5}\,\text{Σ}\,\left(1-\frac{\mathrm{recorded}\,\mathrm{heartbeats}-\mathrm{counted}\,\mathrm{heartbeats}}{\mathrm{recorded}\,\mathrm{heartbeats}}\right)\times 100$$

Time accuracy scores were calculated as the relative difference between the actual time and the time counted. This value was also subtracted from 1 and averaged across all trials to yield a time counting score, such that scores near 100 indicate higher accuracy in time counting. The mathematical formula was, thus, as follows:

$$\frac{1}{5}\,\text{Σ}\,\left(1-\frac{\mathrm{actual}\,\mathrm{time}-\mathrm{counted}\,\mathrm{time}}{\mathrm{actual}\,\mathrm{time}}\right)\times 100$$

### MMSE

The MMSE (Folstein et al., 1975) was used to evaluate cognitive function and exclude patients with dementia. The MMSE is a widely used neuropsychological battery to assess cognitive function and screen for dementia. The components of the MMSE are as follows: (1) orientation, (2) immediate memory, (3) attention and calculation, (4) delayed memory, and (5) language. The total score of the MMSE is 30 points, and the mean score is 27.6 (SD = 1.7) in healthy older adults.

### Image Preprocessing

Structural and functional images were preprocessed, using CONN’s default preprocessing pipeline, in CONN Toolbox version 18.b (Whitfield-Gabrieli and Nieto-Castanon, 2012), implemented in the MATLAB R2016b environment (The MathWorks, Inc., Natick, MA, United States). After the first ten volumes were discarded to allow for magnetic field stabilization, functional scans underwent cerebrospinal fluid (CSF)/white matter (WM) noise removal via the anatomical component-based noise correction procedure (aCompCor), slice time- and motion-correction (realigned), scrubbing, unwarping, co-registration to structural scans, linear detrending but no despiking, normalization to the Montreal Neurological Institute (MNI) atlas space, and spatial smoothing with an 8-mm full width at half maximum (FWHM) Gaussian kernel.

Subject-level gray matter (GM) volume probability maps were derived from T1-weighted images using voxel-based morphometry (VBM). VBM was performed using Statistical Parametric Mapping (SPM12) (Wellcome Trust Centre for Neuroimaging)^1^, and included segmenting individual T1-weighted images into GM volume, WM and CSF using an adaptive Maximum A Posterior technique (Rajapakse et al., 1997), which eliminates the use of tissue priors.

### Statistical Analysis

Functional connectivity analyses were performed using the default FC processing pipeline in the CONN Toolbox version 18.b (Whitfield-Gabrieli and Nieto-Castanon, 2012). In this processing pipeline, using a component-based noise correction method (Behzadi et al., 2007), physiological and other spurious sources of noise were removed together with the movement- and artifact-related covariates. The blood oxygen level dependent (BOLD) signal from the cerebral white matter and ventricles was removed prior to seed-based connectivity analysis using a principal component analysis of the multivariate BOLD signal within each of the masks obtained from the segmented T1-weighted scan (Woodward et al., 2011). The residual BOLD time-series was then band-pass filtered (0.008–0.09 Hz). We generated seed region of interest (ROI) to target ROI connectivity maps for each participant using reproducibly demonstrated SN seeds. The seed ROIs comprised 10-mm diameter spheres at the following anatomical locations and spatial coordinates: bilateral rostral prefrontal cortex (Brodmann’s area (BA) = 10; right, *x* = 32, *y* = 46, *z* = 27; left, *x* = −32, *y* = 45, *z* = 27), ACC (BA = 32, *x* = 0, *y* = 22, *z* = 35), bilateral anterior insular cortex (BA = 13; right, *x* = 47, *y* = 14, *z* = 0; left, *x* = −44, *y* = 13, *z* = 1), and bilateral SMG (BA = 40; right, *x* = 62, *y* = −35, *z* = 32; left, *x* = −60, *y* = −39, *z* = 31). The target ROIs comprised a whole-brain set of 164 ROIs defining the BA (Talairach atlas; Lancaster et al., 2000). The seed ROIs are provided by the CONN toolbox, and represent core topological nodes within the SN. The reasoning for the identification and use of these seeds is described in greater detail by the originators of the CONN toolbox (Whitfield-Gabrieli et al., 2011).

In a group-level analysis, one-sample general linear model analyses were conducted to examine the positive and negative correlations between heartbeat counting accuracy and SN FC, with age, sex, MMSE score, and total GM volume as covariates. A whole-brain height threshold of *p* < 0.001 (uncorrected) was used to identify areas with a significant correlation. A false discovery rate (FDR)-corrected threshold of *p* < 0.05 at this height threshold was applied for all reported clusters.

Group-level independent component analysis (ICA), using the CONN Toolbox version 18.b, was conducted to identify the network of functionally connected brain regions during resting state that may be correlated with interoceptive accuracy scores. This involved the application of the fast ICA algorithm to volumes concatenated across subject and resting state conditions to identify independent spatial components (ICs) and the back-projection of these components to individual subjects, which produced regression coefficients maps representing connectivity between the network and every voxel in the brain. Forty ICs were identified using spatial overlap of suprathreshold areas (Dice coefficient; Rombouts et al., 1998), based on CONN’s default network atlas with ROIs characterizing an extended set of salience (7 ROIs), visual (4 ROIs) network. We selected 40 ICs based on previous research suggesting that ICA results are only affected by the number of ICs when it is smaller than the number of source signals (Ma et al., 2007), in addition to coverage of most signal variance. Forty ICs were included in multiple regressions with interoceptive accuracy scores. For each network, the resulting statistical maps had a voxel threshold of *p* < 0.05 (uncorrected) and cluster threshold of *p* < 0.05 (FDR-corrected). All coordinates reported below refer to peak activations in anatomical MNI space.

In comparison to a previous study in younger adults (Chong et al., 2017), to elucidate which insular subdivision(s) and their connected brain regions are linked to interoceptive accuracy, we further examined the relationship between voxel-wise connectivity maps of each insular subdivision and the heartbeat counting accuracy scores. We first derived eight insular subdivision seeds (both lateral mid-posterior, anterior-ventral, anterior-dorsal, and middle insular) from Kurth et al. (2010). The CONN Toolbox version 18.b performs seed-based analysis by computing the temporal correlation between the BOLD signals from a given seed to all other voxels in the brain. The residual BOLD time-series was then band-pass filtered (0.008–0.09 Hz). A linear regression analysis was conducted to remove signals from the ventricular area and white matter. To minimize the effects of head movement, motion parameters were included in the linear regression analysis. To estimate the strength of an FC, correlation coefficients were computed and converted to *z*-values using Fisher’s r-to-z transformation. Finally, whole-brain voxel-wise regression of each seed-based connectivity map against the heartbeat counting accuracy scores was conducted to identify brain regions connected to the insular subdivisions correlated with heartbeat counting accuracy scores. A whole-brain height threshold of *p* < 0.05 (uncorrected) was used to identify areas with a significant correlation. A FDR-corrected threshold of *p* < 0.05 at this height threshold was applied for all reported clusters.

Spearman’s rank correlation coefficient (ρ) was used to examine correlations among age, sex (with 0 indicating female and 1 indicating male), total MMSE score, heartbeat counting accuracy scores, time counting accuracy scores, and GM volume of set of salience (7 ROIs) and visual network (4 ROIs). Because we cannot hardly say with any finality that sample size is enough for following normal distribution, we calculated Spearman’s rank correlation coefficients (ρ). Moreover, we adjusted the *p*-value using Benjamini and Hochberg’s method (Benjamini and Hochberg, 1995). Data were analyzed using SPSS 25 (IBM Corp., Armonk, NY, United States), with *p* < 0.05 considered to be significant.

## Results

### Behavioral Results

The mean age, sex, total MMSE score, and accuracy scores (for heartbeat and time counting) are listed in Table 1. The heartbeat counting accuracy score ranged between 37.64 and 101.37 (78.96 ± 18.09). Furthermore, the heartbeat counting accuracy score was not significantly correlated with age, sex, MMSE score, or time counting accuracy score (Table 2).

**TABLE 1:** Characteristics for each variable.

	**Mean**	**SD**	**Min**	**Max**
Age	77.30	6.20	61.20	86.90
Sex	19 female		–	–
MMSE	27.96	6.24	22.0	30.0
IA	77.96	18.09	37.64	101.37
TA	103.14	23.49	54.79	156.94

*MMSE, Mini-Mental State Examination; IA, Interoceptive Accuracy; TA, Time counting Accuracy.*

**TABLE 2:** Spearman’s ρ correlation coefficients among variables.

	**Age**	**Sex**	**MMSE**	**IA**	**TA**
age	–	−0.12 (−0.48 ≦ρ≦0.27)	−0.11 (−0.47 ≦ρ≦0.28)	0.03 (−0.35 ≦ρ≦0.41)	0.03 (−0.35 ≦ρ≦0.41)
sex		–	−0.32 (−0.62 ≦ρ≦0.07)	0.46 (0.10 ≦ρ≦0.71)	0.10 (−0.29 ≦ρ≦0.46)
MMSE			–	−0.06 (−0.43 ≦ρ≦0.33)	0.09 (−0.30 ≦ρ≦0.45)
IA				–	−0.08 (−0.45 ≦ρ≦0.31)
TA					–

*MMSE, Mini-Mental State Examination; IA, Interoceptive Accuracy; TA, Time counting Accuracy, 95% confidence intervals in parentheses.*

### Association Between SN FC and Heartbeat Counting Accuracy

**FIGURE 1:**
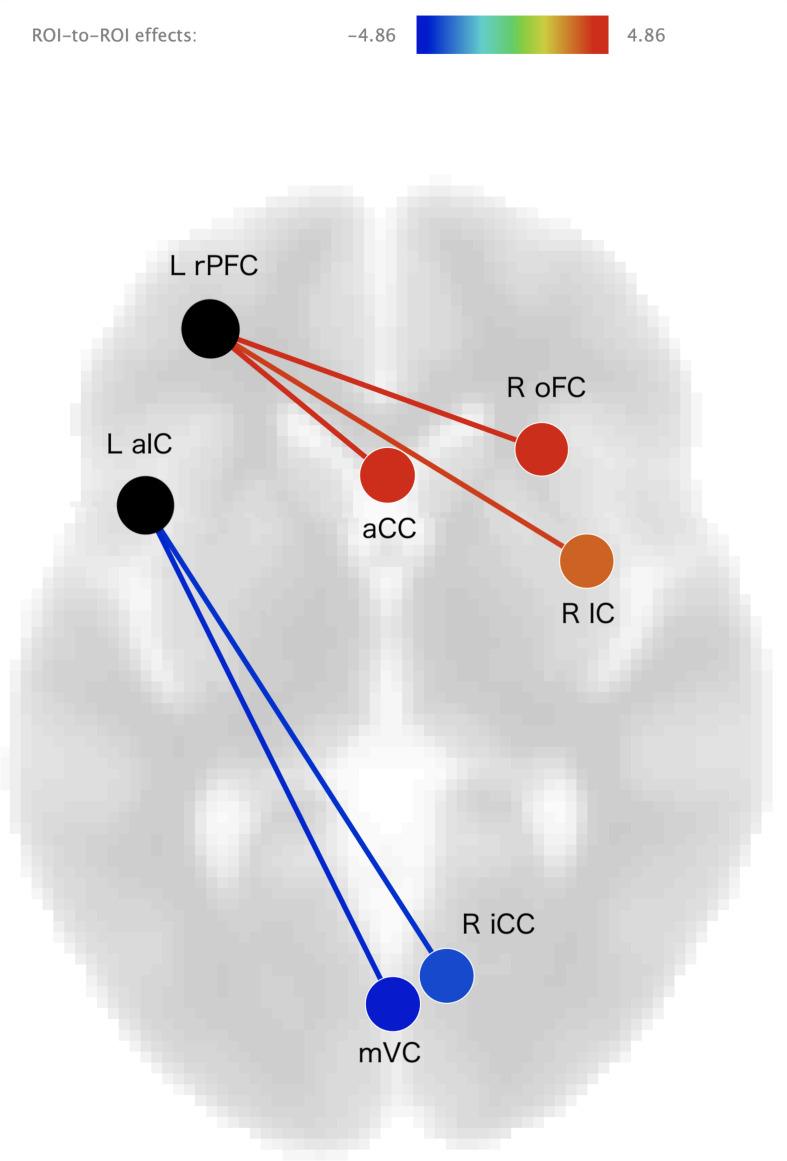
Visual depiction of significant positive correlations (red) and negative correlations (blue) between seed functional connectivity and interoceptive accuracy scores (after statistically accounting for age, sex, MMSE score, and GM volume). Seed regions are labeled in black. L rPFC, left rostral prefrontal cortex; R IC, right insular cortex; R oFC, right orbitofrontal cortex; aCC, anterior cingulate cortex; L aIC, left anterior insular cotex; R iCC, right inter-calcarine cortex; mVC, medial visual cortex; MMSE, Mini-Mental State Examination; GM, gray matter.

**FIGURE 2:**
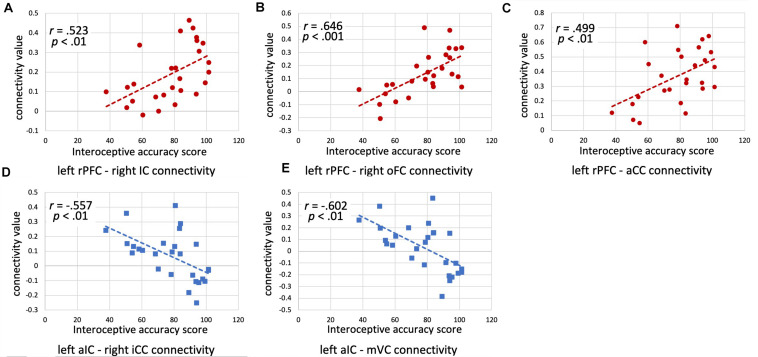
Scatterplots illustrating the correlations between interoceptive accuracy scores and functional connectivity values (z-scores) for each ROI seed-target pair (accounting for age, sex, and MMSE score) are shown. **(A)** Left rPFC–Right IC connectivity; **(B)** Left rPFC–Right oFC connectivity; **(C)** Left rPFC–aCC connectivity; **(D)** Left aIC–Right iCC connectivity; **(E)** Left aIC–mVC connectivity. rPFC, rostral prefrontal cortex; IC, insular cortex; oFC, orbitofrontal cortex; aCC, anterior cingulate cortex; aIC, anterior insular cortex; iCC, inter-calcarine cortex; mVC, medial visual cortex; MMSE, Mini-Mental State Examination.

The results of the ROI-to-ROI analysis are illustrated in Figure 1. We found a significant positive correlation between the heartbeat counting accuracy scores and the FC of the left rostral prefrontal cortical seed with the group-level SN (*F*(6,16) = 4.52, FDR-corrected *p* < 0.05). Moreover, higher heartbeat counting accuracy was associated with increased FC of the left rostral prefrontal cortical seed with the right insular cortex (*t*(21) = 4.86, FDR-corrected *p* < 0.05; Figure 2A), right orbitofrontal cortex (*t*(21) = 4.44, FDR-corrected *p* < 0.05; Figure 2B), and ACC (*t*(21) = 3.89, FDR-corrected *p* < 0.05; Figure 2C), even when controlling the effects of age, sex, MMSE score, and GM volume. Additionally, we found a significant negative correlation between heartbeat counting accuracy and the FC of the left anterior insular cortical seed with the group-averaged SN (*F*(6,16) = 2.04, FDR-corrected *p* < 0.10). Moreover, higher heartbeat counting accuracy was associated with decreased FC of the left anterior insular cortical seed with right intra-calcarine cortices (*t*(21) = −4.20, FDR-corrected *p* < 0.05; Figure 2D), and visual medial cortex (*t*(21) = −4.04, FDR-corrected *p* < 0.05; Figure 2E), even after controlling the effects of age, sex, MMSE score, and total GM volume. Scatterplots depicting the relationship between heartbeat counting accuracy and FC values are shown in Figure 2.

**FIGURE 3:**
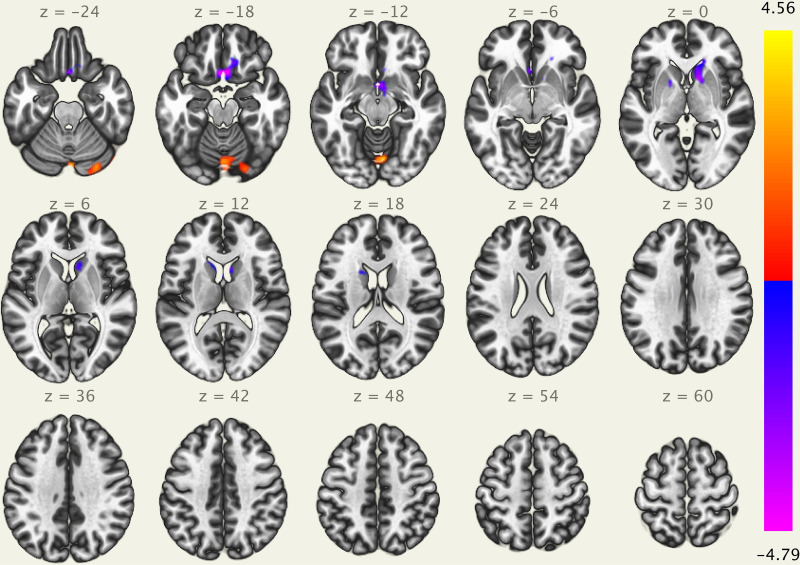
Visual depiction of significant positive correlation (red) and negative correlation (blue) between voxel-based functional connectivity and interoceptive accuracy scores (after statistically accounting for age, sex, MMSE score, and GM volume). Interoceptive accuracy scores show significant positive correlation with the cerebellum (peak voxel = 56, –66, –36), and significant negative correlation with the lateral orbitofrontal cortex (peak voxel = 2, 18, –20) and caudate nucleus (peak voxel = 12, 14, –1); MMSE, Mini-Mental State Examination; GM, gray matter.

The results of the ICA analysis are illustrated in Figure 3. We found a significant positive correlation between the heartbeat counting accuracy scores and the cerebellum (peak voxel = 56, −66, −36; cluster size of 1135 voxels, *t*(21) = 4.56 FDR-corrected *p* < 0.05). Additionally, we found a significant negative correlation between the heartbeat counting accuracy scores and both the lateral orbitofrontal cortex (peak voxel = 2, 18, −20; cluster size of 1231 voxels, *t*(21) = −4.79, FDR-corrected *p* < 0.05) and caudate nucleus (peak voxel = 12, 14, −1; cluster size of 1135 voxels, *t*(21) = −3.90 FDR-corrected *p* < 0.05), even after controlling the effects of age, sex, MMSE score, and GM volume. Moreover, no significant correlations were observed between visual network connectivity and heartbeat counting accuracy scores, or between GM volumes of a set of SN (7 ROIs) and heartbeat counting accuracy scores.

We conducted additional analysis on the correlation between four subdivision(s) of the insular cortex and heartbeat counting accuracy scores. Higher heartbeat counting accuracy was associated with decreased FC of the left middle insular gyrus as seed with the visual medial cortex (*t*(21) = −4.53, FDR-corrected *p* < 0.05), left lingual gyrus (*t*(21) = −4.15, FDR-corrected *p* < 0.05), and right supracalcarine cortex (*t*(21) = −3.91, FDR-corrected *p* < 0.05), even after controlling the effects of age, sex, MMSE score, and total GM volume.

## Discussion

In the present study, we performed an ROI-to-ROI analysis to investigate the relationships between interoceptive accuracy and SN connectivity. Interoceptive accuracy, as assessed by the heartbeat counting task, was positively associated with FC between the left rostral prefrontal cortex and brain regions within the SN, including the right insular cortex, ACC, and right orbitofrontal cortex. In contrast, interoceptive accuracy was negatively associated with FC between the left anterior insular cortex and visual-processing brain regions, including the right intra-calcarine cortices and visual medial cortex. Moreover, ICA analysis showed that interoceptive accuracy was positively associated to cerebellum and negatively associated with both lateral orbitofrontal cortex and caudate nucleus. Therefore, this study revealed that the SN plays an important role in maintaining interoceptive accuracy in older adults.

### Neural Correlates of Interoception

Neural signals for interoception are generated by the transmission of information regarding the physical condition to the brain via the afferent system, with control by the efferent system. The autonomic nervous system, consisting of the sympathetic, parasympathetic, and enteric nervous systems, and visceral afferents, is greatly involved in interoception. Autonomic nervous system output involves several interconnected areas distributed throughout the forebrain and brainstem. The primary forebrain autonomic areas are the insular cortex, ACC, and midcingulate cortex. Especially, the posterior insular cortex receives and integrates interoceptive or bodily sensations. The posterior insula cortex projects to the anterior insula, which integrates interoceptive signals and is involved in the conscious experience of bodily sensation, including the timing of the heartbeat. The subgenual ACC projects to the autonomic areas involved in parasympathetic control of the heart. These visceromotor cortices play a major role in interoception by issuing prediction signals on the expected state of the body based on previous experience; these interoceptive predictions are then compared to current visceral sensations as a mechanism to correct autonomic output (Bennarroch, 2020). In particular, the transmission of information from the right insular cortex to the ACC and orbitofrontal cortex has been observed in young adolescents (Dennis et al., 2014). Furthermore, the report by Craig (2009) hypothesized that subjective perceptions of internal sensations depend on the right anterior insular cortex.

In an fMRI study, Critchley et al. (2004) investigated the neural circuitry underlying performance on a heartbeat discrimination task; the results revealed the right anterior insular cortex as an important region for interoceptive accuracy. Moreover, GM volume in the right anterior insula, orbitofrontal cortex, and midline cerebellum was correlated with interoceptive accuracy. Schulz (2016) conducted a meta-analysis of nine studies and found that heart-focused interoceptive accuracy, and interoceptive accuracy in general, was correlated with activity in the posterior right and left insula, right claustrum, precentral gyrus, and medial frontal gyrus. Thus, interoceptive accuracy, regardless of the type of task, is correlated with task-related activity in the insular cortex and ACC.

Recently, resting state fMRI studies have described the default mode network (DMN), executive control network (ECN), and SN as associated with high-level cognitive processes. Interoceptive-system hubs (e.g., ACC, insular cortex, and amygdala) overlap considerably with the SN (Kleckner et al., 2017). The results of the present study support the hypothesis that better performance on the heartbeat counting task is correlated with greater SN FC in older adults. The rostral prefrontal cortex seed was located in Brodmann’s area 10, which is known to be involved in prospective memory tasks (Okuda et al., 1998), but in recent years, it has also been reported as related to pain (as pain matrix; Peng et al., 2018) and decision-making, in terms of complex information ethics (Sevinc et al., 2017). Sevinc et al. (2017) argued that the SN is implicated in the coordination of executive control and associative processes. Therefore, these results suggest that the rostral prefrontal cortex coordinates with the insular cortex, ACC, and orbitofrontal cortex to maintain interoceptive accuracy in older adults.

### Differences Between Younger and Older Adults in the Neural Correlates of Interoceptive Accuracy

Chong et al. (2017) showed a positive correlation between the heartbeat counting score and SN FC of the right and left posterior insular cortices in healthy young adults, but no correlation was found for SN FC of the ACC. Moreover, no significant negative correlations were detected between the heartbeat counting score and SN FC. Critchley et al. (2001) proposed that changes in bodily states involve two hierarchical processes: a first-order context-independent autonomic representation within the insular and somatosensory cortices, and a second-order context- and experience-dependent representation within the cingulate and ventromedial prefrontal cortices. Thus, the results in Chong et al. (2017) indicate that interoceptive accuracy in young adults is more closely associated with context-independent autonomic representation than with context-dependent representation. In contrast to the study by Chong et al. (2017), the present study revealed a positive correlation between interoceptive accuracy and the ACC FC with the SN, using the same paradigm as that in Chong et al. (2017). Thus, the present study results suggest that context- and experience- dependent representation remains in older adults. Other studies have reported that, in older adults, context-dependent memory retrieval promotes the accuracy of a feeling of knowledge (Thomas et al., 2011), and that a protagonist’s memorized personal experience promotes the accuracy of empathy (Wieck and Kunzmann, 2015).

Moreover, the Embodied Predictive Interoception Coding (EPIC) model (Kleckner et al., 2017) proposes that the interoceptive system has monosynaptic bidirectional connections between the insular cortex and ACC (as well as the dorsal amygdala) to exchange interoceptive predictions and prediction error signals. The EPIC model hypothesizes that the anterior insular cortex and ACC, as visceromotor regions, initiate visceromotor predictions through their cascading connections to brain regions (e.g., hypothalamus) that control the body’s internal milieu; simultaneously, the anterior insular cortex and ACC send information regarding the anticipated sensory consequences of interoceptive predictions to the primary interoceptive cortex (e.g., posterior insular cortex). Moreover, the primary interoceptive cortex receives ascending viscerosensory inputs; simultaneously, the posterior insular cortex sends information regarding the sensory consequences of interoceptive prediction errors to the anterior insular cortex and ACC. To summarize previous studies, context-dependent representation refers to the representation of predictions. Thus, the results of Chong et al. (2017) can be interpreted as showing a lack of representation of interoceptive predictions, which might, thus, explain why there was no correlation between interoceptive accuracy and ACC FC with the SN. Therefore, the existence of an association between interoceptive accuracy and ACC FC with the SN in the current study suggests that older adults maintain context-dependent representations, such as interoceptive predictions.

The results of this study suggest that the neural correlates of interoception accuracy differ between older and younger adults. The present study revealed that the left anterior insular cortex is correlated with regions associated with visual exteroception, such as the visual medial cortex and calcarine cortex, in older adults. Chong et al. (2017) reported no negative correlations between interoceptive accuracy and SN FC in younger adults. Previous studies reported that the anterior insular cortex may function as an important node for integrating information across multiple brain networks, such as the DMN and executive attention network (Uddin et al., 2014). In the present study, participants were asked to gaze at the fixation in the center of the screen via a mirror during the resting state scan. The negative connectivity between left anterior insular cortex and vision-related regions might represent negative feedback on vision-related exteroceptive processing to maintain interoceptive processing.

Previous studies reported that brain function or connectivity is decreased in posterior regions and increased in anterior regions in aging (Davis et al., 2008; Jockwitz et al., 2019; Ren et al., 2019). This phenomenon is defined as the Posterior–Anterior Shift in Aging (PASA) (Davis et al., 2008). The PASA model has been mainly used to explain the change in brain patterns involved in the direct linkage between the PASA phenomenon and the cognitive aging process, as well as cognitive maintenance due to PASA. Previous studies reported that DMN FC is associated with cognitive function, and cognitive fatigue was related to the PASA phenomenon (Jockwitz et al., 2019; Ren et al., 2019). Moreover, Kehoe et al. (2013) examined age-related differences in functional reactivity to viewing pictures with high emotional arousal. In this previous study, older adults showed reduced reactivity in the bilateral occipital and temporal visual cortices, left inferior parietal cortex, and bilateral supplementary motor area compared to that in younger adults. The processing of emotional arousal information is largely associated with the SN; thus, the SN may be affected by the PASA phenomena. Future studies are needed to examine the existence of the PASA phenomenon in the SN.

### Sex Differences in Interoceptive Accuracy

In the present study, there were no significant correlations among interoceptive accuracy, age, and cognitive function because the variances of age and MMSE score were small among the 27 participants. Moreover, the present study did not show a significant correlation between interoceptive accuracy and sex. Several earlier studies reported higher interoceptive accuracy scores in men than in women (Ludwick-Rosenthal and Neufeld, 1985; Montoya et al., 1993; Grabauskaitė et al., 2017). Grabauskaitė et al. (2017) suggested that one of the reasons males perform better than females on interoceptive accuracy tasks is the relevance of the biochemical properties of the heart (men have a larger heart capacity and a stronger heart muscle for systole than do women); thus, men are more likely to feel the heartbeat as a stimulus. While previous studies have focused on younger adults, the present study revealed no sex differences in interoceptive accuracy in older adults. These results indicate that sex differences in interoceptive accuracy may be affected by aging. However, higher interoceptive accuracy in women than in men has been reported in studies on mental disorders such as depression, somatic symptom disorder, and personality disorder (Mussgay et al., 1999) and in those with a small ratio of female participants. The present study had fewer male participants than female participants; since discrepancies in sex differences in interoceptive accuracy depend on the characteristics of the participants, careful consideration is required.

### Time Counting Accuracy

In the present study, there was no significant relationship between heartbeat counting accuracy and time counting accuracy. Time counting accuracy reflects individual differences in timing accuracy, and is used as a control task to examine the validity of the heartbeat counting task (to check whether the counting of seconds is used when counting the heartbeats) (Schandry, 1981). The lack of a significant correlation in the present study suggests that participants did not refer to the number of seconds when counting heartbeats, indicating that the purpose of counting heartbeats was achieved. In addition, we excluded participants who could not perceive their own heartbeat without any cues and those with heartbeat counting accuracy ±2SD or more from the mean, which may have contributed to the validity of the interoceptive counting accuracy.

### Limitations

The present study has some limitations. First, the current study participants were female predominant (70%); thus, future studies are needed to examine the sex difference in interoceptive accuracy in a larger sample size with an equal gender ratio. Second, the current study did not examine age-related differences in the correlation between SN FC and individual differences in interoceptive accuracy. Future studies are needed to directly examine differences between older and younger adults. Third, this current study did not examine a questionnaire for interoception. Future studies are needed to examine the correlations or differences between interoceptive accuracy and awareness in functional brain connectivity.

Additionally, the present study used the heartbeat counting task. The heartbeat counting task is the most commonly utilized method for assessing the perception of heartbeat sensations; however, approximately 40% of participants are reportedly not able to consciously register their heartbeats at all (Khalsa et al., 2009a). Khalsa et al. (2009a) proposed a novel protocol based on the standardized isoproterenol sensitivity test, which involves multiple bolus administrations of isoproterenol. Furthermore, another study (Fittipaldi et al., 2020) examined a convergent multidimensional and multi-feature approach to interoception using a novel interoceptive accuracy index (IA-md) based on heartbeat detection tasks. The authors have reported that the IA-md is associated with the electroencephalogram-derived heart-evoked potential, FC of interoceptive seeds (including bilateral insular cortex, ACC, and postcentral cortex), and performance in an emotional face recognition task. Future studies are warranted to further evaluate measuring methods of interoceptive accuracy to develop a comprehensive view of the functional integrity of interoception.

## Conclusion

The current results provide new insights into the relationship between intrinsic SN FC and individual differences in interoceptive accuracy in older adults. The present study showed that interoceptive accuracy is positively correlated with connectivity within the SN and negatively correlated with connectivity between the left insular cortex and occipital cortex during rest in older adults. These findings suggest that the positive correlation between left rostral prefrontal cortex and the SN and the negative correlation between left insular cortex and vision-related exteroceptive brain regions aid in maintaining interoceptive accuracy. Moreover, in reference to previous results in younger adults (Chong et al., 2017), the observed positive correlation among SN might be a comprehensive interoceptive process by several brain regions in older adults. Moreover, the observed negative correlation between left insular cortex and occipital cortex in the interoceptive process might control exteroceptive information in older adults. These results suggest that the PASA phenomenon occurs in the SN to maintain interoceptive accuracy in older adults.

## Data Availability Statement

The data that support the findings of this study are available from the corresponding author, upon reasonable request.

## Ethics Statement

The studies involving human participants were reviewed and approved by Kyoto Prefectural University of Medicine (ERB-C-853-3). The patients/participants provided their written informed consent to participate in this study.

## Author Contributions

DU: execution of the research project, data collection, statistical analysis, writing the first draft, and manuscript review and critique. TM: execution of the research project, data collection, statistical review and critique, and manuscript review and critique. YK: execution of the research project and manuscript review and critique. NA: execution of the research project, statistical review and critique, and manuscript review and critique. SM and MT: data collection and manuscript review and critique. JN: organization and execution of the research project, statistical review and critique, and manuscript review and critique. All authors read and approved the final manuscript.

## Conflict of Interest

The authors declare that the research was conducted in the absence of any commercial or financial relationships that could be construed as a potential conflict of interest.

## Footnotes

1 http://www.fil.ion.ucl.ac.uk/spm/software/spm12/
